# Single Nucleotide Polymorphisms in *HMGB1* Correlate with Lung Cancer Risk in the Northeast Chinese Han Population

**DOI:** 10.3390/molecules23040832

**Published:** 2018-04-04

**Authors:** Min Jiang, Xuelian Li, Xiaowei Quan, Xiaoying Li, Baosen Zhou

**Affiliations:** 1 Department of Epidemiology, School of Public Health, China Medical University, Shenyang 110122, China; mij9620@163.com (M.J.); xlli@mail.cmu.edu.cn (X.L.); quanxiaowei126@126.com (X.Q.); xyingl0608@163.com (X.L.); 2 Key Laboratory of Cancer Etiology and Prevention (China Medical University), Liaoning Province Department of Education, Shenyang 110122, China

**Keywords:** *HMGB1*, single nucleotide polymorphism, lung cancer, susceptibility, prognosis

## Abstract

Lung cancer is the principal cause of cancer-associated deaths. *HMGB1* has been reported to be associated with tumorigenesis. This study aimed to investigate the relationship between rs1412125 and rs1360485 polymorphisms in *HMGB1* and the risk and survival of lung cancer. 850 cases and 733 controls were included. Logistic regression analysis and survival analysis were performed to investigate the association between SNPs and the risk and survival of lung cancer. Crossover analysis was used to analyze the interaction between SNPs and tobacco exposure. Results indicated that rs1412125 polymorphism was associated with lung cancer risk, especially with the risk of lung adenocarcinoma and small cell lung cancer. Carriers with CT and CC genotypes had a decreased risk of lung cancer (CT + CC vs.TT: adjusted OR = 0.736, *p* = 0.004). Similar results were obtained in the stratification analysis for non-smokers and female population. For rs1360485 polymorphism, AG and GG genotypes could decrease the risk of lung adenocarcinoma and female lung cancer by 0.771-fold and 0.789-fold. However, no significant interaction between polymorphisms and tobacco exposure or association between SNPs and the survival of lung cancer was observed. This study indicated polymorphisms in *HMGB1* may be a novel biomarker for female lung adenocarcinoma risk.

## 1. Introduction

Lung cancer, with its low 5-year survival rate, is one of the most frequently diagnosed tumors [1]. The mechanism of its progression and development is still unclear. Smoking has been recognized as a pivotal environmental risk factor for lung cancer. Carcinogens and their metabolites (i.e., *N*-nitrosamines and polycyclic aromatic hydrocarbons) from cigarette smoke can activate multiple pathways such as cell proliferation, migration and apoptosis involved in tumor promotion and progression [2]. In addition to environmental factors, genetic susceptibility may participate in the development and progression of lung cancer too. To predict the susceptibility and survival of lung cancer, understanding of the genetic mechanisms, including DNA genotyping and heterogeneity as well as the association between the genetic susceptibility and cigarette smoking is increasingly required [3].

High-mobility group box protein 1 (*HMGB1*), located on human chromosome 13q12, is a nuclear protein that was found in mammals [4,5]. *HMGB1* includes two DNA-binding domains (A-box and B-box) and a negatively charged C-terminus and serves as a chromatin structural protein in the cell nucleus and a pro-inflammatory cytokine extracellularly [6,7]. It also involves in the cell survival and death by participating in the apoptosis, immune response as well as the autophagy in tumor progression [8,9,10,11]. In recent studies, *HMGB1* has been proved to be related to the tumor development such as gastric carcinoma [12], colorectal carcinoma [13,14], pancreatic carcinoma [15], glioma [16], thymic epithelial tumors [17], non-Hodgkin lymphoma [18], breast cancer [19], cervical carcinoma [20], lung cancer [21,22,23], hepatocellular carcinoma [24,25] as well as oral cancer [26,27].

**Figure 1:**
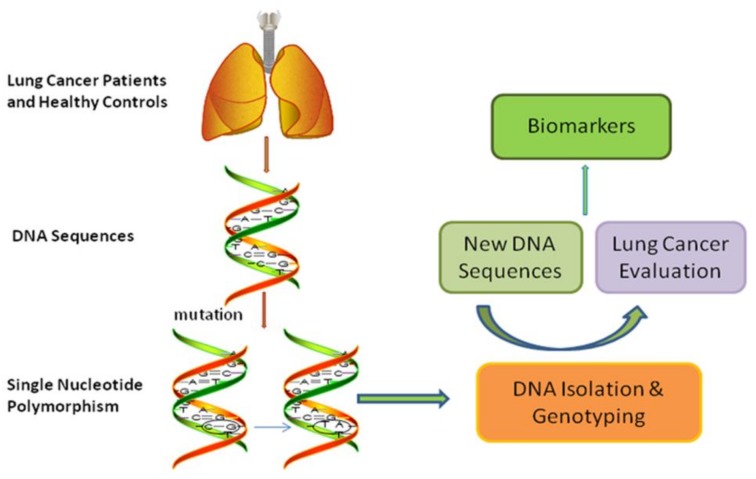
Flow chart of this study to assess the influence of single nucleotide polymorphisms on the risk and survival of lung cancer.

Analysis aiming at comparing distribution frequencies of genotypes among different subgroups is increasingly conducted to assess the susceptibility and survival of cancers [28,29,30]. Previous studies have reported the *HMGB1* polymorphisms might efficiently predict the risk of susceptibility to different cancers such as hepatocellular carcinoma, uterine cervical neoplasia as well as colorectal cancer [31,32,33,34]. Thus we conducted this study to evaluate the association between the two SNPs (rs1412125 and rs1360485) in *HMGB1* and the susceptibility as well as the survival of lung cancer to provide a new biomarker for the diagnosis, susceptibility and prognosis of lung cancer (as shown in Figure 1).

## 2. Results

### 2.1. Study Characteristics

The baseline data of these 850 cases and 733 controls in this present study was summarized, as shown in Table 1. There was no significant difference in the distribution of age (58.63 ± 11.16 years for cases and 57.46 ± 13.18 years for controls, *p* = 0.056) and family history of cancer (*p* = 0.688) between the two subgroups. However, the significant differences existed in the distribution of both smoking status and gender between cases and controls (66.5% and 71.2% non-smokers, 35.38 ± 10.81 packing-years for cases and 29.41 ± 14.76 pack-years for controls, 74.6% and 87.3% female individuals for case and control groups respectively, *p* < 0.001). Therefore, all the further statistical analyses were adjusted for age, gender and smoking status and stratification analysis for gender and smoking status was also conducted to eliminate the influence from their inequality between groups on the following analysis results. Among the 850 cases, there were 507 cases with lung adenocarcinoma (LAD), 212 cases with lung squamous cell carcinoma (LSCC), 106 cases with small cell lung cancer (SCLC) and 25 cases with other histological types. As for the clinical stage, 55 cases were in stage I, 126 cases were in stage II, 488 cases were in stage III-IV and the remaining 181 cases’ information of clinical stage was not obtained. The median follow-up time was 18 months among the 365 cases whose follow-up information was obtained.

### 2.2. Distributions of Genotype Frequency and Associations with Lung Cancer 

Table 2 summarizes the distributions of rs1412125 and rs1360485 alleles and genotypes between cases and controls as well as the associations between genotypes and the susceptibility to lung cancer.

Genotype frequencies of rs1412125 (*p* = 0.529) and rs1360485 (*p* = 0.945) polymorphisms were satisfied to HWE in controls. For the distribution of rs1412125 polymorphism, significant difference existed between TT and CT genotypes that carriers with CT genotype had a 0.744-fold decreased risk of susceptibility to lung cancer than carriers with TT genotype. The distribution of rs1412125 polymorphism was also prominently different in the dominant model between the cases and controls that subjects with CT and CC genotypes had a 0.736-fold decreased risk of susceptibility to lung cancer than those carrying TT genotype. For the allele comparison of rs1360485 polymorphism, G allele decreased the risk for lung cancer by 0.829-fold while different rs1360485 genotypes were not associated with the risk of susceptibility to lung cancer.

### 2.3. Stratified Analysis

Results in Table 3 and Table 4 indicated that rs1412125 polymorphism might have an effect on the risk of susceptibility to LAD and SCLC. Relative to TT genotype carriers, individuals with CT genotype had a 0.764-fold and 0.576-fold decreased risk of LAD and SCLC respectively while for the dominant models individuals who carried CT and CC genotypes had a 0.752-fold and 0.595-fold decreased risk of LAD and SCLC respectively. Among non-smokers, CT genotype decreased the risk for lung cancer by 0.756-fold while carriers with CT and CC genotypes had a 0.750-fold decreased risk of lung cancer. When it came to the female population, carriers with CT genotype had a 0.727-fold decreased risk of lung cancer and carriers with CT and CC genotypes had a 0.725-fold decreased risk. Results of stratified analyses for SNP rs1360485 suggested that AG and GG genotypes could decrease the risk for LAD by 0.771-fold and decrease the risk for female lung cancer by 0.789-fold when the AA genotype was considered as a reference (Tables S1 and S2).

### 2.4. Interaction between SNPs and Tobacco Exposure

Results of the crossover analysis were summarized in Table 5. When compared with non-smokers who carried CT and CC genotypes, TT genotype carriers with tobacco exposure, CT and CC carriers with tobacco exposure and TT genotype carriers without tobacco exposure obtained 3.456-fold, 2.467-fold and 1.399-fold increased risk of susceptibility to lung cancer respectively for rs1412125 polymorphism. Nevertheless, there was no addictive interaction or multiplicative interaction between rs1412125 or rs1360485 polymorphisms and tobacco exposure (Table 6 and Table 7).

### 2.5. Survival Analysis

Results of COX regression analysis demonstrated that no significant association existed between these two SNPs and the survival of lung cancer (Table 8). In addition, no significant difference could be observed in the distribution of rs1412125 or rs1360485 polymorphisms in different clinical stages (Table S3).

## 3. Discussion

Lung cancer with high morbidity and mortality is one of the most malignant cancers around the world. Therefore, reducing the morbidity and mortality becomes an important challenge for the public healthcare. The development and progression of lung cancer contain multiple processes which could be affected by environmental factors and genetic or epigenetic regulations. Genetic mutations involve in the tumorigenesis, cancer progression and prognosis while SNPs could regulate the expression of gene or affect gene’s functions and then alter the phenotypes. 

SNP rs1412125 (−1615T/C), located in −1615 base pairs upstream of *HMGB1*, acts as a transcription repressor to inhibit the transcription process [35,36]. The mutant allele C would lose the inhibition function and result into the overexpression of *HMGB1*. SNP rs1360485 is located in the intron region of *HMGB1*. Although polymorphism rs1360485 could not change the sequence of *HMGB1* protein, it might regulate transcription process of *HMGB1* or other gene. A series of studies have reported the association between polymorphisms rs1412125 or rs1360485 and the risk of cancers such as oral squamous cell carcinoma [27], hepatocellular carcinoma [31], uterine cervical neoplasia [33], colorectal cancer [34] as well as the lung cancer chemotherapy response [37]. Lin et al. conducted a study to verify the association between four SNPs (rs1412125, rs2249825, rs1045411, and rs1360485) in *HMGB1* and the risk of oral squamous cell carcinoma (OSCC). They found that only the rs1045411 polymorphism could affect the risk of OSCC while other three SNPs might not be related to the susceptibility to OSCC [27]. Wu’s study showed an association of SNPs of *HMGB1* with the risk of susceptibility to uterine cervical neoplasia for Taiwanese women. Results indicated that the risk of cervical invasive cancer was 1.85-fold for women with TC and 1.99-fold for women with TC/CC when compared with TT carriers in *HMGB1* rs1412125 polymorphism [33]. The association of *HMGB1* polymorphisms with the risk of colorectal carcinoma was also investigated in a Chinese population. However, there was no significant association between the rs1412125 polymorphism and the risk of susceptibility to colorectal cancer [34]. Wang et al. reported the effects of four *HMGB1* SNPs (rs1412125, rs1045411, rs2249825, and rs1360485) on the susceptibility and development of hepatocellular cancer and results indicated that carriers with TT genotype had a higher risk of distant metastasis compared with individuals carrying at least one C allele for rs1412125 polymorphism [31]. Significant associations were found between rs1412125 polymorphism and the platinum-based chemotherapy response in both genotypic and recessive models. The same result was also observed in the subgroup of cases aged over 55 years in additive and recessive as well as the genotypic models [37].

In this current study, we estimated the association between SNPs (rs1412125 and rs1360485) in *HMGB1* and the susceptibility of lung cancer among 850 cases and 733 controls. There are two main reasons why we did not study the other two SNPs (rs2249825 and rs1045411): (1) the results of our previous GWAS study indicated that an association between the two SNPs (rs1412125 and rs1360485) and the risk of lung cancer might exist. However, the other two SNPs (rs2249825 and rs1045411) did not exist in the GWAS loci of lung cancer; (2) according to the results of the ensembl database (the website of the ensembl database: http://www.ensembl.org/index.html) and the previous articles, a strong linkage disequilibrium exists not only between rs1360485 and rs1045411 but also between rs1360485 and rs2249825 [27,38,39]. It means that we just need to study only one SNP to explore the association between the SNP and the lung cancer risk and the analysis result can represent all these three SNPs. Among these three SNPs, rs1360485 is the most common one so that we selected the rs1360485 as a tagSNP rather than studying all of these three SNPs. Therefore, only rs1412125 and rs1360485 were contained in this study. Results indicated that rs1412125 polymorphism in *HMGB1* was associated with the risk of susceptibility to lung cancer, especially with the risk of susceptibility to LAD and SCLC. Subjects who carried TT genotype had higher risk of LAD and SCLC than those who carried CT and CC genotypes. In the stratification analysis for non-smokers and female population, the same results were also obtained. For rs1360485 polymorphism, the G allele could reduce the risk for lung cancer and results of stratified analyses demonstrated that AG and GG genotypes could reduce the risk for LAD as well as female lung cancer compared with the AA genotype. Results of our study were inconsistent with the former one conducted by Hu et al. [39]. They examined four *HMGB1* SNPs (rs2249825, rs1360485, rs1045411 and rs1412125) in 190 lung cancer cases and 187 healthy controls. Results indicated CT or TT + CT genotypes of rs1045411 polymorphism could reduce the risk of lung cancer and the T/C/G haplotypes of rs1045411, rs2249825 and rs1360485 also decreased the risk of lung cancer by 0.486-fold while no significant association was found between rs1412125 polymorphism and the risk of susceptibility to lung cancer. Their results were inconsistent with ours and the reasons might come from the following different aspects: (a) the different sample sizes. This current study enrolled 850 cases and 733 controls while only 190 patients and 187 controls participated in the genotypic frequency analysis for Hu’s study; (b) the different areas. Our study was conducted in the northeast of China while the east of China was selected by Hu et al. Although all the participants were Chinese Han population for both these two studies, the results could still be inconsistent as a result of some unknown environmental factors due to different areas; (c) the different inclusion criteria. Hu’s findings might have been influenced by inclusion of cases that had been diagnosed as lung cancer patients before their study began, which might result into the prevalence-incidence bias. This present study only enrolled the newly diagnosis so that it could avoid this problem.

For this study, some limitations still existed. Firstly, the sample size was relatively small, especially in the stratification analysis. Secondly, all the participants for this study were from the northeast of China and this might cause the bias of the results. Therefore, studies with large sample sizes and more diversified population are needed to confirm these results.

In spite of the defects above, there were some advantages in this present study. One strength was that this was a multi-center and large sample-size study, which could enhance the reliability of the results. In addition, all the cases were newly diagnosed as lung cancer patients to prevent the prevalence-incidence bias so that the results of this study was more credible. Finally, not only the pooled analysis but also the stratified analysis was conducted according to the smoking status, genders as well as the pathological types to provide a detailed analysis of the association between the single nucleotide polymorphism in *HMGB1* and the lung cancer risk.

In summary, this study provided evidence that polymorphisms in table (rs1412125 and rs1360485) might alter the individual susceptibility to lung cancer. However, future larger studies with different ethnic and area populations are still required to confirm these current findings.

## 4. Materials and Methods

### 4.1. Study Subjects

This is a molecular epidemiologic study of lung cancer in Shenyang, located in northeast China. 850 cases and 733 controls were included in our hospital-based case-control study. All of the cases were recruited from the First Affiliated Hospital of China Medical University, the Fourth Affiliated Hospital of China Medical University as well as Liaoning Cancer Hospital (between January 2010 and January 2014). Inclusions of case group as follows: (a) newly diagnosed histologically as lung cancer patients; (b) without any chemotherapy or radiotherapy. Exclusion criteria included the previous cancer or metastasized cancer from the different cancer. Meanwhile, 733 healthy controls without lung cancer were recruited from the medical examination center of the same hospital. Controls were frequency matched to cases on age (±5 years). All of the participants were unrelated ethnic Han Chinese population.

Some data was collected including age, gender, smoking status, clinical stage as well as the pathologic types. Individuals who smoked less than 100 cigarettes for the entire lifetime were considered as non-smokers. In the meantime, the cases were followed up for at least two years. The study was conducted in accordance with the Declaration of Helsinki, and the protocol was approved by the Institutional Review Board of China Medical University and each subject had signed the informed consent form.

### 4.2. DNA Isolation Genotyping

The genomic DNA of every participant was extracted from the venous blood sample with the phenol-chloroform method. The SNP genotyping was conducted by the 7500 Fast Real-Time PCR system (Applied Biosystems, Foster City, CA, USA) and the PCR Taqman probes and primers were designed by the same company (assay ID C___8690889_10 for rs1412125 and C___8690872_10 for rs1360485). The reaction condition of quantitative real-time PCR (qRT-PCR) was heating to 95 °C for 10 min, 30 s at 92 °C and 1 min at 60 °C for 47 cycles. To control the quality, 5% samples from the subgroups of both cases and controls were randomly chosen to analysis again and results were consistent with the former ones. The ancestral and derived Alleles of rs1412125 polymorphism are T and C respectively while the ancestral and derived Alleles of rs1360485 polymorphism are A and G respectively. Therefore, the wild homozygous of rs1412125 and rs1360485 SNPs is TT and AA respectively, and the mutant homozygous of the two SNPs is CC and GG respectively. The heterozygote is CT and AG respectively.

### 4.3. Statistical Analysis

The student’s *t*-test was carried out in continuous variables while Pearson’s χ^2^ test was conducted in the categorical variables. A goodness-of-fit χ^2^ test was conducted to investigate the Hardy-Weinberg equilibrium (HWE) in control group. Odds Ratios (ORs) and their 95% Confident Intervals (95% CIs) were computed by unconditional logistic regression analysis after adjustment for age, gender as well as the smoking status to assess the association between the two SNPs and the risk of susceptibility to lung cancer. Interaction between SNPs and tobacco exposure was evaluated by crossover analysis. The multiplicative interaction was estimated by OR and its 95% CI with the unconditional logistic regression model. Relative Excess Risk due to Interaction (RERI), Synergy Index(S) and Attributable Proportion due to Interaction (AP) were used to estimate the addictive interaction. If the 95% CI of RERI and AP did not contain 0 and the 95% CI of S did not contain 1, the statistical difference was significant and there might be interactions between SNPs and the tobacco exposure [40]. Hazard Ratios (HRs) and the 95% CIs were computed by COX regression analysis to evaluate the potential association between these two SNPs and the survival of lung cancer. All of the statistical analyses were two-sided and carried out by SPSS software (vision 22.0, IBM SPSS, lnc. Chicago, IL, USA). Criterion of the statistical significance was *p* < 0.05.

## 5. Conclusions

Results of this current study suggested that polymorphisms in *HMGB1* showed an association with the risk of lung cancer in non-smokers and female population and it could be used as a biomarker for the risk of lung cancer, especially for the risk of LAD and SCLC. However, studies with larger sample sizes might be needed to validate these findings and the biological functions of these two polymorphisms in lung cancer will be explored in the future.

## Figures and Tables

**Table 1:** Demographics of lung cancer patients and control.

Variables		Cases (%)	Controls (%)	*p*-Value
Age (year)	mean ± S.D.	58.63 ± 11.16	57.46 ± 13.18	0.056
Gender	male	216 (25.4%)	93 (12.7%)	<0.001
	female	634 (74.6%)	640 (87.3%)	
Smoking *	never	565 (66.5%)	522 (71.2%)	<0.001
	yes	241 (28.3%)	82 (11.2%)	
	packing-years	35.38 ± 10.81	29.41 ± 14.76	0.011
Family history of cancer *	yes	66 (12.2%)	61 (13.1%)	0.688
	no	473 (87.8%)	405 (86.9%)	
Histology	LAD	507 (59.6%)		
	LSCC	212 (24.9%)		
	SCLC	106 (12.5%)		
	others	25 (3.0%)		
Clinical stage	Ⅰ	55 (6.5%)		
	Ⅱ	126 (14.8%)		
	Ⅲ	332 (39.0%)		
	Ⅳ	156 (18.4%)		
	unknown	181 (21.3%)		
Survival time *	M (P_25_~P_75_)	18 (11.0~32.0)		
Status	alive	54 (14.8%)		
	dead	311 (85.2%)		

LAD, lung adenocarcinoma; LSCC, lung squamous cell carcinoma; SCLC, small cell lung cancer; * There were missing values.

**Table 2:** Distribution of rs1412125 and rs1360485 and lung cancer risk.

Genotype	Cases (%)	Controls (%)	OR(95% CI) *	*p*-Value	HWE
rs1412125					0.529
TT	511 (60.1%)	396 (54.0%)	1		
CT	296 (34.8%)	290 (39.6%)	0.744(0.599, 0.924)	0.008	
CC	43 (5.1%)	47 (6.4%)	0.684 (0.434, 1.077)	0.101	
Dominant model					
TT	511 (60.1%)	396 (54.0%)	1	0.004	
CT + CC	339 (39.9%)	337 (46.0%)	0.736 (0.597, 0.906)		
Recessive model					
TT + CT	807 (94.9%)	686 (93.6%)	1	0.247	
CC	43 (5.1%)	47 (6.4%)	0.769 (0.493, 1.200)		
Allele model					
T allele	1318 (77.5%)	1082 (73.8%)	1	0.102	
C allele	382 (22.5%)	384 (26.2%)	0.769 (0.561, 1.053)		
rs1360485					0.945
AA	579 (68.1%)	464 (63.3%)	1		
AG	245 (28.8%)	238 (32.5%)	0.836 (0.668, 1.046)	0.117	
GG	26 (3.1%)	31 (4.2%)	0.667 (0.381, 1.168)	0.157	
Dominant model					
AA	579 (68.1%)	464 (63.3%)	1	0.066	
AG + GG	271 (31.9%)	269 (36.7%)	0.816 (0.658, 1.013)		
Recessive model					
AA + AG	824 (96.9%)	702 (95.8%)	1	0.219	
GG	26 (3.1%)	31 (4.2%)	0.706 (0.405, 1.231)		
Allele model					
A allele	1403 (82.5%)	1166 (79.5%)	1	0.047	
G allele	297 (17.5%)	300 (20.5%)	0.829 (0.689, 0.998)		

HWE, Hardy-Weinberg equilibrium; OR, Odd ratio; 95% CI, 95% Confidence Interval; *OR was adjusted by age, gender and smoking.

**Table 3:** Stratification analysis of rs1412125 polymorphisms and risk of lung cancer.

Histology	Genotype	Cases (%)	Controls (%)	OR(95% CI) *	*p*-Value
LAD	TT	302 (59.6%)	396 (54.0%)	1	
	CT	180 (35.5%)	290 (39.6%)	0.764 (0.598,0.975)	0.031
	CC	25(4.9%)	47 (6.4%)	0.680 (0.405, 1.143)	0.146
	Dominant model				
	TT	302 (59.6%)	396 (54.0%)	1	0.018
	CT + CC	205 (40.4%)	337 (46.0%)	0.752 (0.595, 0.951)	
	Recessive model				
	TT + CT	482 (95.1%)	686 (93.6%)	1	0.287
	CC	25 (4.9%)	47 (6.4%)	0.759 (0.456, 1.262)	
LSCC	TT	125 (59.0%)	396 (54.0%)	1	
	CT	75 (35.4%)	290 (39.6%)	0.807 (0.560, 1.162)	0.249
	CC	12 (5.6%)	47 (6.4%)	0.706 (0.333, 1.498)	0.364
	Dominant model				
	TT	125 (59.0%)	396 (54.0%)	1	0.191
	CT + CC	87 (41.0%)	337 (46.0%)	0.792 (0.558, 1.123)	
	Recessive model				
	TT + CT	200 (94.3%)	686 (93.6%)	1	0.487
	CC	12 (5.7%)	47 (6.4%)	0.770 (0.368, 1.610)	
SCLC	TT	69 (65.1%)	396 (54.0%)	1	
	CT	31 (29.2%)	290 (39.6%)	0.576 (0.364, 0.911)	0.018
	CC	6 (5.7%)	47 (6.4%)	0.719 (0.291, 1.777)	0.475
	Dominant model				
	TT	69 (65.1%)	396 (54.0%)	1	0.019
	CT + CC	37 (34.9%)	337 (46.0%)	0.595 (0.386, 0.918)	
	Recessive model				
	TT + CT	100 (94.3%)	686 (93.6%)	1	0.787
	CC	6 (5.7%)	47 (6.4%)	0.885 (0.363, 2.154)	

LAD, lung adenocarcinoma; LSCC, lung squamous cell carcinoma; SCLC, small cell lung cancer; OR, Odd ratio; 95% CI, 95% Confident Interval; * OR was adjusted by age, gender and smoking.

**Table 4:** Stratification analysis of rs1412125 polymorphisms and risk of lung cancer.

Variables	Genotype	Cases (%)	Controls (%)	OR (95%CI) *	*p*-Value
Smoking-no	TT	339 (60.0%)	276 (52.9%)	1	
	CT	199 (35.2%)	215 (41.2%)	0.756 (0.589, 0.971)	0.028
	CC	27 (4.8%)	31 (5.8%)	0.709 (0.413, 1.217)	0.213
	Dominant model				
	TT	339 (60.0%)	276 (52.9%)	1	0.019
	CT + CC	226 (40.0%)	246 (47.1%)	0.750 (0.589, 0.954)	
	Recessive model				
	TT + CT	538 (95.2%)	491 (94.1%)	1	0.393
	CC	27 (4.8%)	31 (5.9%)	0.794 (0.467, 1.349)	
Smoking-yes	TT	141 (58.5%)	41 (50.0%)	1	
	CT	87 (36.1%)	34 (41.5%)	0.756 (0.441, 1.296)	0.308
	CC	13 (5.4%)	7 (8.5%)	0.518 (0.192, 1.399)	0.194
	Dominant model				
	TT	141 (58.5%)	41 (50.0%)	1	0.198
	CT + CC	100 (41.5%)	41 (50.0%)	0.713 (0.427, 1.192)	
	Recessive model				
	TT + CT	228 (94.6%)	75 (91.5%)	1	0.272
	CC	13 (5.4%)	7 (8.5%)	0.582 (0.222, 1.529)	
Gender-male	TT	127 (58.8%)	49 (52.7%)	1	
	CT	77 (35.6%)	36 (38.7%)	0.848 (0.492, 1.462)	0.553
	CC	12 (5.6%)	8 (8.6%)	0.492 (0.176, 1.373)	0.175
	Dominant model				
	TT	127 (58.8%)	49 (52.7%)	1	0.350
	CT + CC	89 (41.2%)	44 (47.3%)	0.781 (0.466, 1.311)	
	Recessive model				
	TT + CT	204 (94.4%)	85 (91.4%)	1	0.208
	CC	12 (5.6%)	8 (8.6%)	0.526 (0.193, 1.431)	
Gender-female	TT	384 (60.6%)	341 (54.2%)	1	
	CT	219 (34.5%)	254 (39.7%)	0.727 (0.573, 0.922)	0.009
	CC	31 (4.9%)	39 (6.1%)	0.709 (0.427, 1.176)	0.182
	Dominant model				
	TT	384 (60.6%)	347 (54.2%)	1	0.006
	CT + CC	250 (39.4%)	293 (45.8%)	0.725 (0.577, 0.911)	
	Recessive model				
	TT + CT	603 (95.1%)	601 (93.9%)	1	0.388
	CC	31 (4.9%)	39 (6.1%)	0.804 (0.489, 1.321)	

OR, Odd ratio; 95% CI, 95% Confident Interval; * OR was adjusted by age, gender and smoking.

**Table 5:** Crossover analysis of interaction between SNPs and tobacco exposure in northeast Chinese population.

Genotype	Smoking	Cases (%)	Controls (%)	OR(95% CI) *	*p*-Value
rs1412125					
CT + CC	—	226 (28.0%)	246 (40.7%)	1	
TT	—	339 (42.1%)	276 (45.7%)	1.339 (1.053, 1.703)	0.017
CT + CC	+	100 (12.4%)	41 (6.8%)	2.467 (1.571, 3.875)	<0.001
TT	+	141 (17.5%)	41 (6.8%)	3.465 (2.222, 5.401)	<0.001
rs1360485					
AG + GG	—	188 (23.3%)	192 (31.8%)	1	
AA	—	377 (46.8%)	330 (54.6%)	1.169 (0.911, 1.500)	0.220
AG + GG	+	70 (8.7%)	31 (5.2%)	2.150 (1.297, 3.566)	0.003
AA	+	171 (21.2%)	51 (8.4%)	3.173 (2.074, 4.855)	<0.001

OR, Odd ratio; 95% CI, 95% Confident Interval; * OR was adjusted by age and gender.

**Table 6:** Addictive interaction between SNPs and tobacco exposure in northeast Chinese population.

Genotype	Measure *	Estimate *	Lower *	Upper *
rs1412125	RERI	0.008	−0.538	0.554
	AP	0.008	−0.550	0.567
	S	0.750	0.000	101,194,884.087
rs1360485	RERI	0.010	−0.560	0.580
	AP	0.010	−0.573	0.594
	S	0.720	0.000	36,041,230.440

RERI, Relative Excess Risk due to Interaction; AP, Attributable Proportion due to Interaction; S, Synergy Index; * All value was adjusted by age and gender.

**Table 7:** Logistic model of multiplicative interaction between SNPs and tobacco exposure.

Genotype	Variables	OR (95% CI) *	*p*-Value
rs1412125	rs1412125	0.747 (0.587, 0.950)	0.017
	smoking	2.587 (1.672, 4.004)	<0.001
	interaction	0.954 (0.546, 1.666)	0.868
rs1360485	rs1360485	0.855 (0.666, 1.098)	0.220
	smoking	2.714 (1.809, 4.071)	<0.001
	interaction	0.792 (0.442, 1.419)	0.433

OR, Odd ratio; 95% CI, 95% Confident Interval; * OR was adjusted by age and gender.

**Table 8:** The association between SNPs and survival of lung cancer.

SNPs	Cases	Deaths	MST (Months)	HR (95% CI) *	*p*-Value
rs1412125					
TT	218	185	18	1	
CT	129	110	19	0.934 (0.733, 1.189)	0.579
CC	18	16	12	1.267 (0.759, 2.115)	0.366
rs1360485					
AA	255	216	19	1	
AG	102	88	19	0.877 (0.668, 1.152)	0.347
GG	8	7	12	0.924 (0.693, 1.252)	0.589

HR, Hazard Ratio; 95% CI, 95% Confident Interval; * HR was adjusted by age, gender and smoking.

## Supplementary Materials

The Supplementary Materials are available online.

## Abbreviations

*HMGB1*	High-mobility group box protein 1
SNP	single nucleotide polymorphism
NSCLC	non-small cell lung cancer
qRT-PCR	quantitative real-time Polymerase Chain Reaction
min	minute
HWE	Hardy-Weinberg equilibrium
OR	Odd ratio
95% CI	95% Confident Interval
RERI	Relative Excess Risk due to Interaction
AP	Attributable Proportion due to Interaction
S	Synergy Index
HR	Hazard Ratio
LAD	lung adenocarcinoma
LSCC	lung squamous cell carcinoma
SCLC	small cell lung cancer
OSCC	oral squamous cell carcinoma
