# Supplementary material for: Single Nucleotide Polymorphisms in HMGB1 Correlate with Lung Cancer Risk in the Northeast Chinese Han Population
Source: Molecules. 2018 Apr 4;23(4):832. doi: 10.3390/molecules23040832 (PMC6017634; doi:10.3390/molecules23040832)
Supplement: Supplementary file 1 [file molecules-23-00832-s001.zip › Table S3 the association between SNPs and clinical stage of lung cancer.docx]

**Table S3 the association between SNPs and clinical stage of lung cancer**

| **SNPs** |  |  | **Stage** |  | **χ^2^** | ***P*-value** |
| --- | --- | --- | --- | --- | --- | --- |
|  | **Ⅰ** | **Ⅱ** | **Ⅲ** | **Ⅳ** |  |  |
| rs1412125 |  |  |  |  |  |  |
| TT | 29 | 72 | 209 | 92 | 4.947 | 0.51 |
| CT | 25 | 48 | 108 | 58 |  |  |
| CC | 1 | 6 | 15 | 6 |  |  |
| rs1360485 |  |  |  |  |  |  |
| AA | 35 | 78 | 236 | 106 | 8.492 | 0.204 |
| AG | 20 | 45 | 86 | 45 |  |  |
| GG | 0 | 3 | 10 | 5 |  |  |
