# Supplementary material for: Single Nucleotide Polymorphisms in HMGB1 Correlate with Lung Cancer Risk in the Northeast Chinese Han Population
Source: Molecules. 2018 Apr 4;23(4):832. doi: 10.3390/molecules23040832 (PMC6017634; doi:10.3390/molecules23040832)
Supplement: Supplementary file 1 [file molecules-23-00832-s001.zip › Table S2 stratification analysis of rs1360485 polymorphisms and risk of lung cancer.docx]

**Table S2 stratification analysis of rs1360485 polymorphisms and risk of lung cancer**

| **Variables** | **genotype** | **Cases (%)** | **Controls (%)** | **OR (95%CI)*** | ***P*-value** |
| --- | --- | --- | --- | --- | --- |
| Smoking-no | AA | 377 (66.7%) | 330 (63.2%) | 1 |  |
|  | AG | 172 (30.4%) | 172 (33.0%) | 0.880 (0.680,1.139) | 0.331 |
|  | GG | 16 (2.9%) | 20 (3.8%) | 0.696 (0.354,1.367) | 0.293 |
|  | Dominant model |  |  |  |  |
|  | AA | 377 (66.7%) | 330 (63.2%) | 1 | 0.239 |
|  | AG+GG | 188 (33.3%) | 192 (36.8%) | 0.861 (0.670,1.105) |  |
|  | Recessive model |  |  |  |  |
|  | AA+AG | 549(97.2%) | 502 (96.2%) | 1 | 0.348 |
|  | GG | 16 (2.8%) | 20 (3.8%) | 0.726 (0.371,1.418) |  |
| Smoking-yes | AA | 171 (71.0%) | 51 (62.2%) | 1 |  |
|  | AG | 62 (25.7%) | 26 (31.7%) | 0.733 (0.416,1.291) | 0.283 |
|  | GG | 8 (3.3%) | 5 (6.1%) | 0.494 (0.153,1.592) | 0.237 |
|  | Dominant model |  |  |  |  |
|  | AA | 171 (71.0%) | 51 (62.2%) | 1 | 0.182 |
|  | AG+GG | 70 (29.0%) | 31 (37.8%) | 0.694 (0.406,1.186) |  |
|  | Recessive model |  |  |  |  |
|  | AA+AG | 233 (96.7%) | 77 (93.9%) | 1 | 0.300 |
|  | GG | 8 (3.3%) | 5 (6.1%) | 0.543 (0.171,1.725) |  |
| Gender-male | AA | 148 (68.5%) | 61 (65.6%) | 1 |  |
|  | AG | 62 (28.7%) | 27 (29.0%) | 1.091 (0.610,1.953) | 0.769 |
|  | GG | 6 (2.8%) | 5 (5.4%) | 0.478 (0.131,1.746) | 0.264 |
|  | Dominant model |  |  |  |  |
|  | AA | 148 (68.5%) | 61 (65.6%) | 1 | 0.966 |
|  | AG+GG | 68 (31.5%) | 32 (34.4%) | 0.988 (0.568,1.718) |  |
|  | Recessive model |  |  |  |  |
|  | AA+AG | 210 (97.2%) | 88 (94.6%) | 1 | 0.242 |
|  | GG | 6 (2.8%) | 5 (5.4%) | 0.465 (0.129,1.678) |  |
| Gender-female | AA | 431 (68.0%) | 403 (63.0%) | 1 |  |
|  | AG | 183 (28.9%) | 211 (33.0%) | 0.795 (0.622,1.017) | 0.068 |
|  | GG | 20 (3.1%) | 26 (4.0%) | 0.731 (0.395,1.353) | 0.318 |
|  | Dominant model |  |  |  |  |
|  | AA | 431 (68.0%) | 403 (630%) | 1 | 0.049 |
|  | AG+GG | 203 (32.0%) | 237 (37.0%) | 0.789 (0.622,0.999) |  |
|  | Recessive model |  |  |  |  |
|  | AA+AG | 614 (96.8%) | 614 (95.9%) | 1 | 0.439 |
|  | GG | 20 (3.2%) | 26 (4.1%) | 0.786 (0.427,1.447) |  |

OR, Odd ratio; 95% CI, 95% Confident Interval;*OR was adjusted by age, gender and smoking.
