# Supplementary material for: Single Nucleotide Polymorphisms in HMGB1 Correlate with Lung Cancer Risk in the Northeast Chinese Han Population
Source: Molecules. 2018 Apr 4;23(4):832. doi: 10.3390/molecules23040832 (PMC6017634; doi:10.3390/molecules23040832)
Supplement: Supplementary file 1 [file molecules-23-00832-s001.zip › Table S1 stratification analysis of rs1360485 polymorphisms and risk of lung cancer.docx]

**Table S1 stratification analysis of rs1360485 polymorphisms and risk of lung cancer**

| **Histology** | **Genotype** | **Cases (%)** | **Controls (%)** | **OR(95% CI)*** | ***P*-value** |
| --- | --- | --- | --- | --- | --- |
| LAD | AA | 349 (68.8%) | 464 (63.3%) | 1 |  |
|  | AG | 144 (28.4%) | 238 (32.5%) | 0.795 (0.616,1.025) | 0.077 |
|  | GG | 14 (2.8%) | 31 (4.2%) | 0.590 (0.304, 1.142) | 0.117 |
|  | Dominant model |  |  |  |  |
|  | AA | 349 (68.8%) | 464 (63.3%) | 1 | 0.038 |
|  | AG+GG | 158 (31.2%) | 269 (36.7%) | 0.771 (0.603, 0.986) |  |
|  | Recessive model |  |  |  |  |
|  | AA+AG | 493 (97.2%) | 702 (95.8%) | 1 | 0.172 |
|  | GG | 14 (2.8%) | 31 (4.2%) | 0.633 (0.328, 1.221) |  |
| LSCC | AA | 143 (67.5%) | 464 (63.3%) | 1 |  |
|  | AG | 61 (28.8%) | 238 (32.5%) | 0.914 (0.625, 1.334) | 0.640 |
|  | GG | 8 (3.7%) | 31 (4.2%) | 0.889 (0.363, 2.175) | 0.797 |
|  | Dominant model |  |  |  |  |
|  | AA | 143 (67.5%) | 464 (63.3%) | 1 | 0.614 |
|  | AG+GG | 69 (32.5%) | 269 (36.7%) | 0.911 (0.633, 1.310) |  |
|  | Recessive model |  |  |  |  |
|  | AA+AG | 204 (96.2%) | 702 (95.8%) | 1 | 0.845 |
|  | GG | 8 (3.8%) | 31 (4.2%) | 0.915 (0.377, 2.222) |  |
| SCLC | AA | 72 (67.9%) | 464 (563.3%) | 1 |  |
|  | AG | 30 (28.3%) | 238 (32.5%) | 0.819 (0.517, 1.297) | 0.395 |
|  | GG | 4(3.8%) | 31 (4.2%) | 0.804 (0.271, 2.386) | 0.695 |
|  | Dominant model |  |  |  |  |
|  | AA | 72 (67.9%) | 464 (63.3%) | 1 | 0.369 |
|  | AG+GG | 34 (32.1%) | 269 (36.7%) | 0.817(0.526, 1.270) |  |
|  | Recessive model |  |  |  |  |
|  | AA+AG | 102 (96.2%) | 702 (95.8%) | 1 | 0.779 |
|  | GG | 4 (3.8%) | 31 (4.2%) | 0.857 (0.292, 2.520) |  |

LAD, lung adenocarcinoma; LSCC, lung squamous cell carcinoma; SCLC, small cell lung cancer; OR, Odd ratio; 95% CI, 95% Confident Interval; *OR was adjusted by age, gender and smoking.
